# A Case Report Examining a Contraindication for Mechanical Thrombectomy in the Setting of a Large Vessel Occlusion and a Concurrent Contralateral Intracranial Hemorrhage

**DOI:** 10.7759/cureus.13956

**Published:** 2021-03-17

**Authors:** Stephen Albano, Mary Grace Bacani, Arthur Omuro

**Affiliations:** 1 Neurosurgery, Desert Regional Medical Center, Palm Springs, USA; 2 Intensive Care Unit, Desert Regional Medical Center, Palm Springs, USA; 3 Neurology, Palomar Medical Center, Escondido, USA; 4 Neurology, Scripps Memorial Hospital, Encinitas, USA

**Keywords:** stroke, ischemic stroke, intracerebral hemorrhage, thrombectomy, contraindications

## Abstract

An acute ischemic stroke occurring contralateral to a hemorrhagic stroke is an uncommon occurrence that presents unique challenges. Hemorrhages have classically been described as a contraindication for mechanical thrombectomy. However, the natural course of a large vessel occlusion with or without decompressive hemicraniectomy is associated with significant morbidity and mortality. This paper investigates the origin of the contraindication for mechanical thrombectomy, the natural history of large vessel occlusion, risks of craniectomy, and risks of mechanical thrombectomy. Given the likelihood of poor outcomes without intervention, mechanical thrombectomy could be considered in select individuals, but future studies into the natural course of contralateral ischemic and hemorrhagic strokes would better guide management.

## Introduction

Strokes is the number one cause of adult disability and the fourth leading cause of death in the United States with costs in 2009 both direct and indirect totaling around $68.9 billion [1]. Strokes impact approximately seven million individuals in the United States, about 3% of the adult population [1]. However, not all strokes are created equal. Approximately 87% of strokes are ischemic with the remaining being hemorrhagic [1-3]. Mortality rates associated with ischemic strokes are approximately 57.4% with a subset of those with proximal vessel occlusion in the anterior circulation having a 60-80% mortality in 90 days or inability to regain functional independence [3,4]. Acute ischemic stroke care has evolved over time with studies proving the benefits of acute intervention, such as intravenous thrombolysis and endovascular thrombectomy which can significantly improve a patient’s functional outcome. The following is a case that demonstrates the nuance in a broad guideline that merits further investigation.

## Case presentation

**Figure 1:**
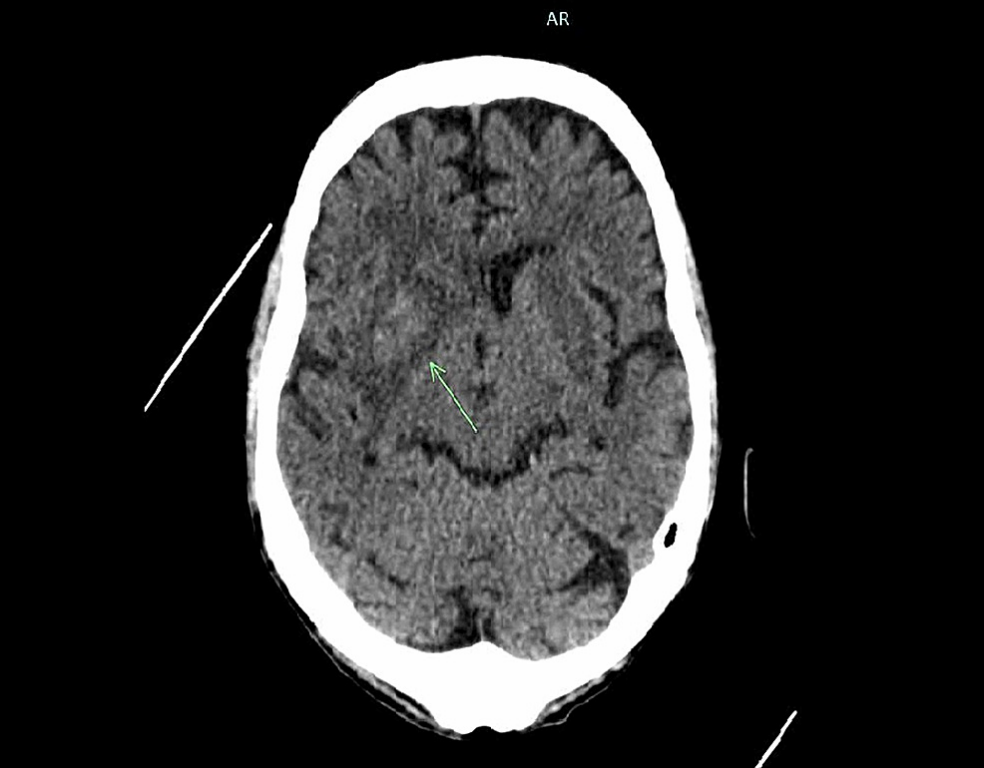
CT non-contrast The arrow indicates an intraparenchymal hemorrhage in the right basal ganglia.

**Figure 2:**
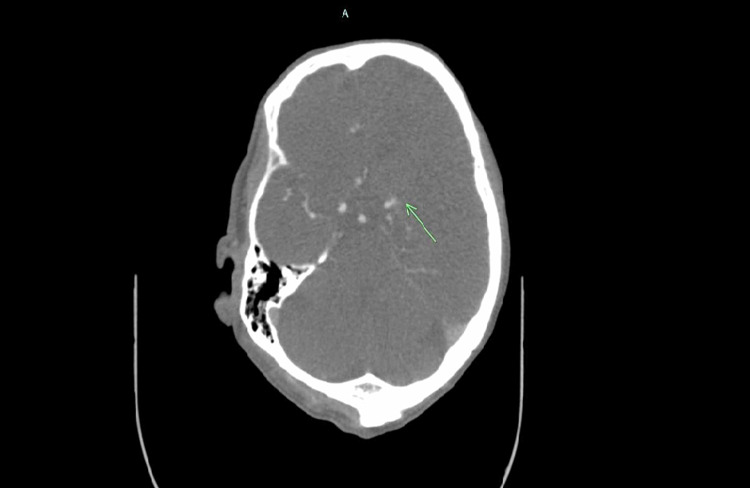
CT angiogram of the head with contrast The arrow indicates proximal left middle cerebral artery occlusion.

**Figure 3:**
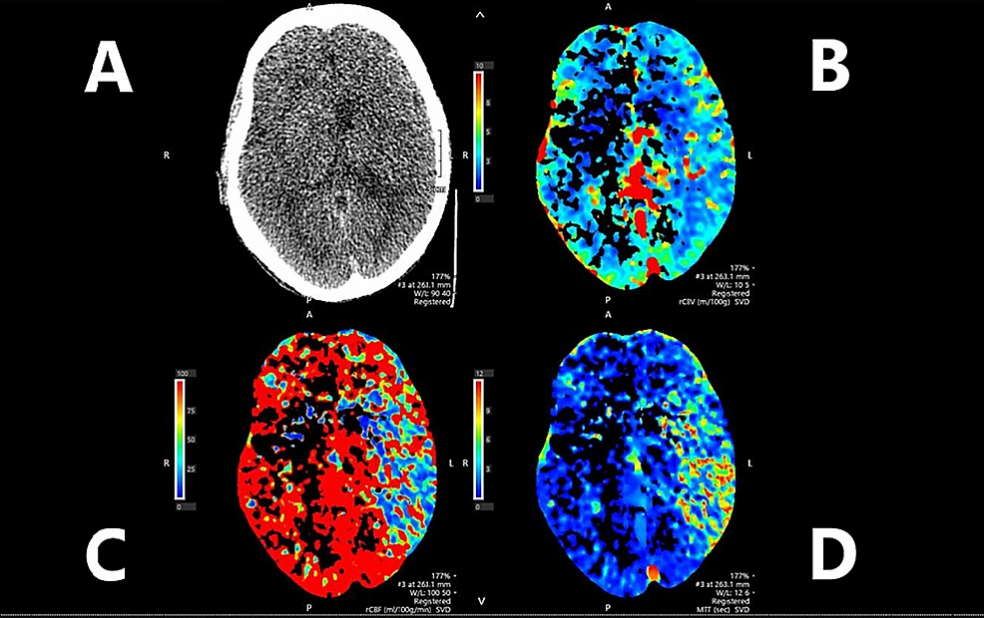
CT perfusion Non-contrast CT head (A), cerebral blood volume (B) showing relatively preserved blood volume, cerebral blood flow (C) showing reduced blood flow, and mean transit time (D) showing increased time within the left cerebral hemisphere.

An 80-year-old male presented from an acute rehab facility for a change in mental status. The patient was recovering at acute rehab following a femoral fracture that was surgically repaired two weeks prior. The last known well was approximately two days prior to the presentation based on a phone call where the patient sounded confused. Staff at the facility noted that on the day prior to presentation, the patient had decreased oral intake but was not aphasic. On the day of the presentation, he was found unresponsive. Upon arrival to the emergency department, the patient was intubated due to Glasgow Coma Scale 3. CT head without contrast revealed a right basal ganglia intracerebral hemorrhage associated with peri-hematoma edema (Figure 1). A follow-up CT angiogram with the contrast of the head however showed a left middle cerebral artery occlusion (Figure 2). With an unreliable timeline of symptoms (stuttering transient ischemic attack vs completed stroke), CT perfusion was performed and demonstrated a left middle cerebral artery territory perfusion mismatch (Figure 3) indicating ischemia and potentially salvageable tissue. Mechanical thrombectomy was not offered due to contralateral hemorrhage. The stroke progressed from ischemia to infarction of the left middle cerebral artery territory with subsequent cerebral edema, midline shift, and herniation. The patient was placed on comfort measures at the direction of the family to coincide with the patient's wishes and expired the following day.

## Discussion

Concurrent contralateral ischemia and hemorrhage

Concurrent contralateral ischemic and hemorrhagic strokes are an uncommon occurrence. A review of the literature revealed prior case reports of patients with contralateral hemorrhage and ischemic lacunar infarcts who recovered with minimal residual deficits following medical management [5]. In these cases, mechanical thrombectomy is not indicated since there is no large vessel occlusion amenable to intervention. However, the role and indications for mechanical thrombectomy in the setting of hemorrhagic stroke with contralateral large vessel occlusion are not well established.

Current guidelines to not perform mechanical thrombectomy in the setting of hemorrhage are based on studies in which hemorrhage was considered exclusion criteria for the study [6-9]. Therefore to our knowledge, no studies are examining the use of mechanical thrombectomy in patients with intracranial hemorrhage. Intuitively we understand that reperfusion of territory with hemorrhage could lead to worsening hemorrhage, especially with the use of heparin which is required for the procedure. However, it is unclear whether or not mechanical thrombectomy should be withheld in a patient with a large vessel occlusion on the basis of a contralateral intracranial hemorrhage.

Natural history of ischemic stroke

In order to guide decision-making regarding concurrent contralateral intracerebral hemorrhage and large vessel occlusions, the natural history of a large vessel occlusion ischemic stroke needs to be better understood. Currently, the literature describes six-month mortality rates in large vessel occlusions at approximately 26.2% [10]. Another study described proximal vessel occlusion in the anterior circulation with a 60-80% 90-day mortality or inability to regain functional independence despite treatment with intravenous alteplase [4]. These numbers are specific towards patients who meet the criteria for intravenous thrombolytics. However, in patients with contralateral hemorrhage, tissue plasminogen activator would not be administered, and as a result, likely incur higher rates of mortality and functional dependence.

The natural history of a large vessel occlusion without intervention would imply a large volume of infarction and therefore should include consideration for decompressive hemicraniectomy. Hemicraniectomy reduced 12-month mortality by nearly 50%, those undergoing medical management demonstrated a 78% mortality, and those with surgery and medical management demonstrated a 29% mortality [10]. Regardless of mortality, hemicraniectomy does not re-perfuse tissue distal to large vessel occlusion and therefore would not improve function attributed to this territory. This should play a role in determining the risks and benefits of performing a mechanical thrombectomy in a patient with a contralateral hemorrhage versus a hemicraniectomy.

Risks of decompressive craniectomy

Risks of decompressive craniectomy can reach 53.9% with one in ten patients needing additional medical or neurosurgical intervention because of a complication [11]. Additional risks include expansion of pre-existing hemorrhagic components in 58% of patients, however, expansion of contralateral hematoma has not been commonly reported in stroke patients [11]. Infarcts progressed to hemorrhage in about 23.7% of malignant stroke patients undergoing craniectomy [11]. Herniation despite craniectomy ranges up to 25% [11]. Prevalence of cerebrospinal fluid leak or fistula can reach 6.3%, with 10% superficial wound infection, meningitis and ventriculitis reaching 4%, 49% with seizures within one-week post hemicraniectomy, and subdural hygromas in 12.5% of patients with hemicraniectomy for stroke. Hydrocephalus can range from 0.7 to 86% with the syndrome of the trephined reaching an overall prevalence of 10% [11].

Risks of mechanical thrombectomy

Risks of mechanical thrombectomy occur in about 11% of the patients. 12 Risks include approximately 5% intracranial hemorrhage post-procedure, 2% risk of embolization, 2% risk of vessel dissection, 3% risk of vasospasm of access vessel, 2% risk of stent dislocation, and 5% risk of stent occlusion [12]. Some of these risks could be mitigated by the use of stent retrievers or aspiration thrombectomy to avoid stent-associated complications. Also, the conversion from an ischemic to hemorrhagic stroke could simplify management in that ischemic and hemorrhagic pathologies have seemingly different objectives in trying to minimize bleeding while optimizing perfusion to any salvageable tissue. This straddling of objectives does not have clear guidelines or evidence leaving management highly experiential with questionable outcomes.

Contraindications for mechanical thrombectomy

Contraindications for mechanical thrombectomy are not clear cut. Current contraindications are experiential or based on inclusion and exclusion criteria from initial studies determining the benefit of mechanical thrombectomy. The MR CLEAN study by Berkhemer et al. defined inclusion criteria to include age greater than 18 years old, National Institutes of Health Stroke Scale (NIHSS) greater than or equal to 2, CT or MRI ruling out intracranial hemorrhage [13]. Exclusion criteria included blood pressure greater than 180/110 mmHg, blood glucose greater than 22.2 mmol/L, platelets less than 40 x 109/L, INR greater than 3.0, and IV alteplase dose exceeding 0.9mg/kg or 90mg [13]. The EXTEND-IA study by Campbell et al., REVASCAT by Jovin et al., and SWIFT PRIME by Saver et al. defined similar inclusion and exclusion criteria, however of note, consistent across these trials, intracranial hemorrhage excluded a patient from these studies [6,7,14]. These inclusion and exclusion criteria were based on theoretical risks of the intervention of study worsening patient status. Intuitively the exclusion of patients with a pre-existing intracerebral hemorrhage makes sense. However, when intracerebral hemorrhage is used as a broad contraindication, then the lack of evidenced-based clarity can preclude patients like the one described in this report from receiving possible mechanical thrombectomy.

Future directions

The natural history of a large vessel occlusion with contralateral intracranial hemorrhage is difficult to describe due to the relative rarity and description in the literature. The case presented here described an 80-year-old male who expired on day two with no tissue plasminogen activator, mechanical thrombectomy, or hemicraniectomy. Another case report of a patient with uncontrolled hypertension and atrial fibrillation with a large right thalamic hematoma was found to have a left internal carotid artery infarct expired on the second day of admission as a consequence of herniation despite anti-edema and antihypertensive medications [15].

With the expected infarction of brain tissue distal to a large vessel occlusion in the setting of a contralateral hemorrhage, risks of morbidity and mortality of non-surgical management or craniectomy should be weighed against those of mechanical thrombectomy. Exclusion from mechanical thrombectomy because of the presence of a contralateral hemorrhage may represent withholding life-saving measures. More studies are needed to identify the natural history of this uncommon occurrence.

## Conclusions

With the expected infarction of parenchymal tissue distal to a large vessel occlusion in the setting of a contralateral hemorrhage, risks of morbidity and mortality of non-surgical management or craniectomy should be weighed against those of mechanical thrombectomy. Current contraindications for mechanical thrombectomy are based on initial benefit studies’ exclusion criteria. Exclusion from mechanical thrombectomy because of the presence of a contralateral hemorrhage may represent with-holding of potentially life-saving measures. More studies are needed to clarify intracerebral hemorrhage as a contraindication to address patient nuances as demonstrated in this case. 
